# Evaluation of high-resolution *In Vivo* MRI for longitudinal analysis of endochondral fracture healing in mice

**DOI:** 10.1371/journal.pone.0174283

**Published:** 2017-03-23

**Authors:** Melanie Haffner-Luntzer, Fabian Müller-Graf, Romano Matthys, Yvonne Hägele, Verena Fischer, René Jonas, Alireza Abaei, Florian Gebhard, Volker Rasche, Anita Ignatius

**Affiliations:** 1 Institute of Orthopedic Research and Biomechanics, University Medical Center Ulm, Ulm, Germany; 2 Department of Traumatology, Hand-, Plastic-, and Reconstructive Surgery, University Medical Center Ulm, Ulm, Germany; 3 RISystem, Davos, Switzerland; 4 Core Facility Small Animal MRI, University Medical Center Ulm, Ulm, Germany; University of Zaragoza, SPAIN

## Abstract

Mice are extensively used for experimental bone-healing studies. However, there are few established nondestructive *in vivo* techniques for longitudinal fracture-healing analysis in mice, including *in vivo* micro-computed tomography (μCT) and radiography. Importantly, none of the established methods can discriminate between non-mineralized fibrous tissue and cartilage in the soft fracture callus. Therefore, the objective was to establish high-resolution *in vivo* magnetic resonance imaging (MRI) for the longitudinal assessment of soft callus formation during bone healing in mice. C57BL/6J mice received a femur osteotomy stabilized using an external fixator and were randomly assigned to five groups. Group 1 mice were scanned three times longitudinally during fracture healing using an optimized MRI scanning protocol to establish an algorithm to characterize the different fracture-callus tissues. Mice of groups 2–4 were scanned once on day 10, 14 or 21, respectively, euthanized after scanning and their femurs subjected to *ex vivo* μCT and histomorphometric analysis to compare the data assessed by MRI with μCT and histology. Control group 5 mice were not scanned. After 28 days, mice of groups 1 and 5 were euthanized and the fracture-healing outcome was evaluated by bending-test, μCT and histology to determine whether the repeated anesthesia, handling and the MRI measurements themselves influenced fracture healing. The callus-tissue values determined by MRI were mostly comparable to those obtained by μCT and histomorphometric analysis. However, at time points characterized by small relative bone or cartilage areas, MRI measurements were weakly comparable to histomorphometric data, possibly due to the inferior spatial resolution. Importantly, at the early and intermediate phases of healing, cartilage and fibrous-tissue values obtained by MRI were highly accurate. Furthermore, repeated anesthesia, handling and MRI scans did not impact bone healing. Therefore, we demonstrated the feasibility of high-resolution *in vivo* MRI for longitudinal assessment of soft callus formation during murine endochondral fracture healing.

## Introduction

Most fractures heal by endochondral bone formation. This process, termed secondary fracture healing, follows a characteristic sequence of tissue differentiation events. The early fracture callus predominantly consists of granulation and fibrous tissues, which are subsequently replaced by cartilage tissue in the intermediate phase of bone healing. During ongoing callus maturation, cartilage is replaced by bony tissue, leading to stable bridging of the fracture gap [1, 2]. The fracture-healing process is influenced by numerous factors, including age, gender, fracture fixation, nutrition, pharmacological therapy and genetic variations, as observed clinically as well as confirmed by murine fracture-healing studies [3]. Disturbed healing becomes visible by alterations in the callus tissue composition in both humans and mice. In particular, the amount of cartilage and fibrous tissue in the fracture callus provides important information on the early bone-healing progress [4].

In preclinical fracture-healing studies using mice, the fracture-callus composition is mostly assessed by micro-computed tomography (μCT) and histomorphometry. However, both methods exhibit significant disadvantages. While μCT analysis is the gold standard for mineralized-tissue visualization, it cannot clearly discriminate between the non-mineralized fibrous and cartilage tissues [5]. Additionally, the relatively high radiation burden of *in vivo* μCT may disturb bone metabolism during repeated examinations [6]. Histomorphometry, on the other hand, is the gold standard for fibrous tissue and cartilage quantification. However, this method is less accurate because of the two-dimensional (2D) evaluation of the fracture callus. Furthermore, the preparation of histological sections requires euthanasia of the mice, not allowing repeated examinations in the same mouse during the healing process. Currently, there is no established method for longitudinal *in vivo* evaluation of the different tissues in the fracture callus in mice. Magnetic resonance imaging (MRI) provides excellent soft-tissue contrast [7, 8] and may be well suited to noninvasively identify soft tissue and cartilage in the fracture callus. However, there are no studies reported testing *in vivo* MRI during endochondral fracture healing in mice. Previous work includes *post mortem* MRI in mice with articular fractures [9] and *in vivo* MRI to monitor intramembranous bone-defect healing [10]. Both studies showed promising results, despite limited spatial resolution and tissue contrast.

The objective of this study was to establish high-resolution *in vivo* MRI for the longitudinal assessment of endochondral fracture healing in mice. We first aimed to establish algorithms for the characterization of the different tissues in the fracture callus. We next correlated the data obtained by MRI with that obtained by μCT and histomorphometry. Finally, we aimed to answer the question whether repeated anesthesia, mouse handling and MRI measurements disturbed the fracture-healing process.

## Materials & methods

### Experimental design and surgery

All animal experiments complied with international regulations for the care and use of laboratory animals and were approved by the regional regulatory authorities (No. 1250, Regierungspräsidium Tübingen, Germany). The animal research facilities of Ulm University provided 12-week-old male C57BL/6J mice. The mice were maintained in groups of two to five animals per cage on a 14-h light and 10-h dark circadian rhythm with water and food provided *ad libitum*. The fracture procedure was published previously [11–13]. Briefly, all mice were anesthetized using isoflurane and received a standardized osteotomy at the midshaft of the right femur using an oscillating micro saw (RISystem, Davos, Switzerland). The osteotomy was stabilized using an external fixator (axial stiffness of 3.0 N/mm) and specifically designed ceramic pins (RISystem) to avoid metal artifacts in the MR images. The animals were randomly assigned to five groups. Mice of group 1 were scanned three times longitudinally during fracture healing to establish algorithms for characterization of the different tissues in the fracture callus. Mice of groups 2–4 (n = 5 for each time point per group) were scanned once on day 10, 14, or 21, respectively, and euthanized directly after the scanning procedure. Femurs were subjected to μCT scanning and decalcified histology to compare the results obtained by MRI, μCT and histology. Mice of the control group 5 did not undergo any scanning procedure. After 28 days, animals of groups 1 and 5 (n = 6–7 per group) were euthanized and the fracture healing-outcome was evaluated by non-destructive 3-point bending test, *ex vivo* μCT and decalcified histology to identify any impact of the repeated anesthesia, mouse handling and MRI examinations on the healing process.

### MRI protocol

**Fig 1:**
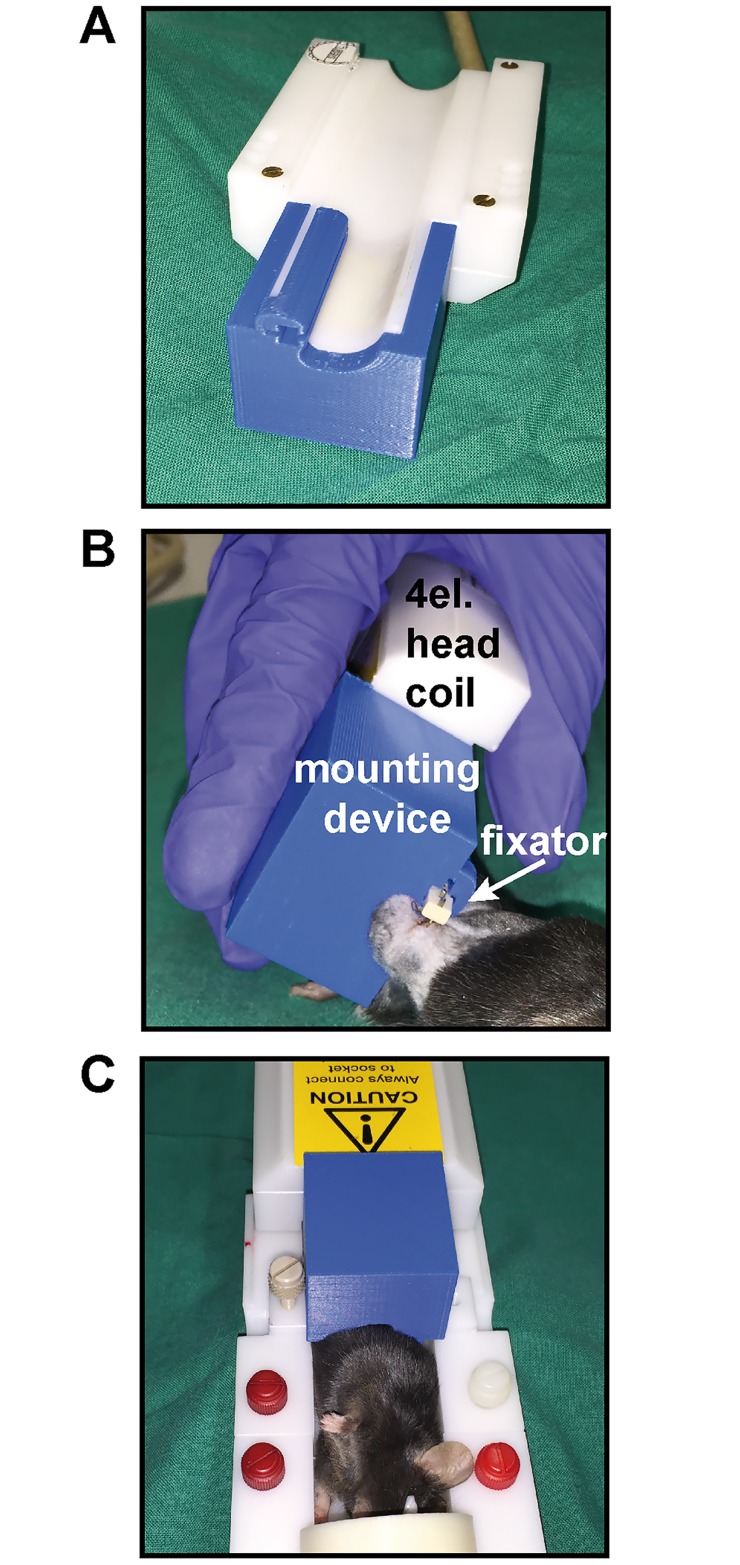
Custom-made mounting device for insertion of the external fixator during MRI scans. A) The mounting device (blue) is plugged on the MRI 4el. head coil (white). B) The external fixator at the right femur of the mouse is inserted into the relief of the mounting device prior to the scanning procedure. C) Mouse plugged into the rack for MRI scan.

All MRI data was acquired using a dedicated high-field small-animal MRI system (BioSpec 117/16, Bruker Biospin, Ettlingen, Germany). For reproducible positioning and immobilization of the femur during scanning, a custom-made fixation device for the external fixator (Fig 1) was developed, which was rigidly attached to the four-element head coil.

The MR data acquisition geometry was aligned with the femur bone, orthogonally to the screws. Data was acquired by applying a proton-density fat-suppressed multi-slice TSE sequence (PD-TSE). Acquisition parameters were: echo/repetition time TE = 5.8 ms/TR = 2500 ms, resolution Δr = 52×52×350 μm^3^, field-of-view fov = 20×20 mm^2^, and bandwidth Δ*ω* = 150 KHz. Total acquisition time for 22 slices was 36 min.

The bone volume in the whole fracture callus between the two inner pinholes was quantified by semi-automatic three-dimensional (3D) segmentation using Avizo software (FEI software, ThermoFisher Scientific, Hillsboro, USA). Callus area as well as cartilage, bone and fibrous tissue fraction quantification was performed by manual segmentation of the different tissues in the two central longitudinal slices through the callus using Osirix software (Pixmeo SARL, Bernex, Switzerland). 3D images were generated using Avizo software. For thresholding of the different tissues, signal intensity values were normalized to the value of mature cortex. Bony tissue (including mature cortex, trabecular bone and bony callus tissue) was segmented within the range of 1–3.3 normalized signal intensity, bone marrow/fibrous tissue within the range of 3.4–5.4, and cartilaginous callus tissue within the range of 5.5–6.2 normalized signal intensity.

### Biomechanical testing

Biomechanical testing of femurs of mice from groups 1 and 5 was performed using a nondestructive 3-point-bending test as described previously [11]. Briefly, after removal of the fixator, a bending load of maximum 4 N was applied on top of the cranio-lateral callus side. Flexural rigidity of the bones was calculated using the slope of the load-deflection curve.

### μCT scans

Fractured femurs were fixed in 4% paraformaldehyde and analyzed by μCT (Skyscan 1172, Kontich, Belgium) yielding an isotropic voxel resolution of 8^3^ μm^3^ (50 kV, 200 mA). Analysis of the bone parameters was performed using Skyscan software (NRecon, DataViewer, CTAn). Two volumes of interest (VOIs) were determined for μCT analysis. VOI 1 covered the whole fracture callus between the two inner pinholes and was used to compare the μCT and MRI data from groups 2–4. VOI 2 covered the endosteal and periosteal callus in the fracture gap and was used to analyze fractured femurs from groups 1 and 5. Apparent bone mineral density was assessed using two phantoms with a defined density of hydroxyapatite (250 and 750 mg/cm^3^) within each scan. To determine the bone volume (BV), a global threshold of 642 mg hydroxyapatite/cm^3^ according to Morgan et al. [5] was used to distinguish between mineralized and non-mineralized tissues. 3D visualizations of the bones were generated using CTAn/CTVol software (Skyscan).

### Histomorphometry of decalcified femora

Femurs were subjected to decalcified histology using 20% ethylenediaminetetraacetic acid (pH 7.2–7.4) for 10–12 days and embedded in paraffin after dehydration in an ascending ethanol series. For groups 1 and 5, central longitudinal sections with a thickness of 7 μm were prepared and stained using Safranin O for tissue quantification. For groups 2–4, serial 7-μm longitudinal sections were cut and stained using Safranin O. Histological sections were registered to the two analyzed MRI slices. Tissue quantification was performed using Metamorph image analysis software (Leica, Wetzlar, Germany). MRI and histomorphometric analyses were performed by two independent investigators.

### Statistics

Sample size of groups 1 and 5 was calculated based on a previous fracture healing study for the main outcome parameter bone volume ratio in the fractured femur (power: 80%, alpha = 0.05) [14]. Data were tested for normal distribution by Shapiro-Wilk test (SPSS statistics, IBM, Ehningen, Germany). In groups 1 and 5, fracture healing outcome parameters were tested for significant differences using Student’s t-test, p<0.05. The following parameters from the fractured femurs were compared between groups 2–4: 3D bone volume (MRI vs. μCT), 2D total callus area and relative bone, cartilage and fibrous tissue areas (MRI vs. histology). The values obtained were analyzed for significant differences between the methods using Student’s t-test, p<0.05. Correlations of the datasets were determined by Pearson’s correlation analysis (Pearson’s correlation coefficient r and coefficient of determination r^2^) and tested for significance of correlation, p<0.05.

## Results

### Longitudinal assessment of callus development during fracture healing using MRI

**Fig 2:**
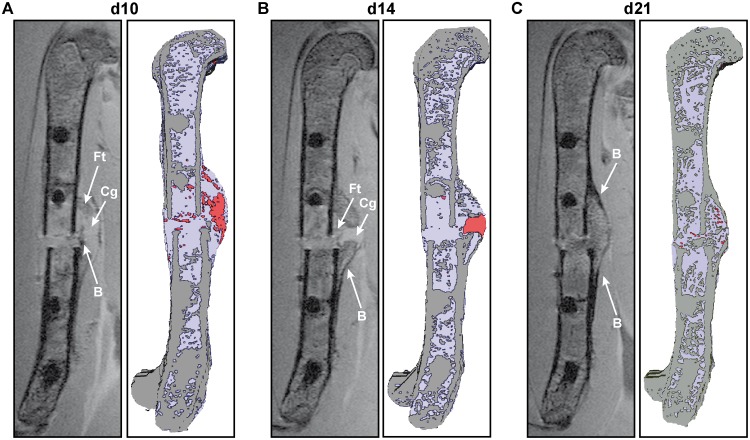
Longitudinal assessment of callus development during fracture healing. 2D MRI slices and 3D reconstructions of the fractured femur from one mouse are shown A) from day 10, B) from day 14 and C) from day 21 after fracture. In the 3D reconstruction images, bone is displayed in grey, cartilage in red and fibrous tissue and bone marrow in light purple. Ft = fibrous tissue; Cg = cartilage; B = bony callus.

Longitudinal scanning of the mice of group 1 revealed that the callus was clearly distinguishable from the surrounding tissue because of a hypo-intense signal from the periosteum at all time points. There were very small, very intense areas surrounding the ends of the fractured cortices from day 10 until day 21, which did not change during the healing process. These are most likely susceptibility artifacts due to the transition from bone to soft tissues. Furthermore, there were hyper-intense areas in the middle of the fracture callus at days 10 and 14. The size of these areas peaked at day 10, decreased until day 14 and were almost absent by day 21. Therefore, we hypothesized that these areas represent cartilage tissue. A slight difference in the signal intensity of the cartilage tissue at the growth plate and the callus was observed, which is likely caused by the coil sensitivity pattern. Hypo-intense areas were observed at day 14 in the periphery of the callus, spreading through the whole callus by day 21. Therefore, we hypothesized that these areas were bony callus tissue. The signal intensity from mature bone (cortex) was lower than that from the newly formed bone in the fracture callus. Only on day 21 was the peripheral bone in the fracture callus as hypo-intense as the mature cortex, indicating final mineralization in parts of the fracture callus. However, the differences in signal intensity were subtle, therefore we did not distinguish between low and high mineralized bone in the fracture callus. There were iso-intense areas in the fracture callus with a grey value between the hyper-intense value of “cartilage” and the hypo-intense value of “new bone”. These areas displayed the same intensity as the bone marrow. Therefore, we hypothesized that these areas were fibrous tissue. Based on these algorithms, we segmented the different tissues in the fracture callus semi-automated by grey-value thresholding and generated 3D images (Fig 2).

### Comparison of 3D data obtained by μCT and MRI

**Fig 3:**
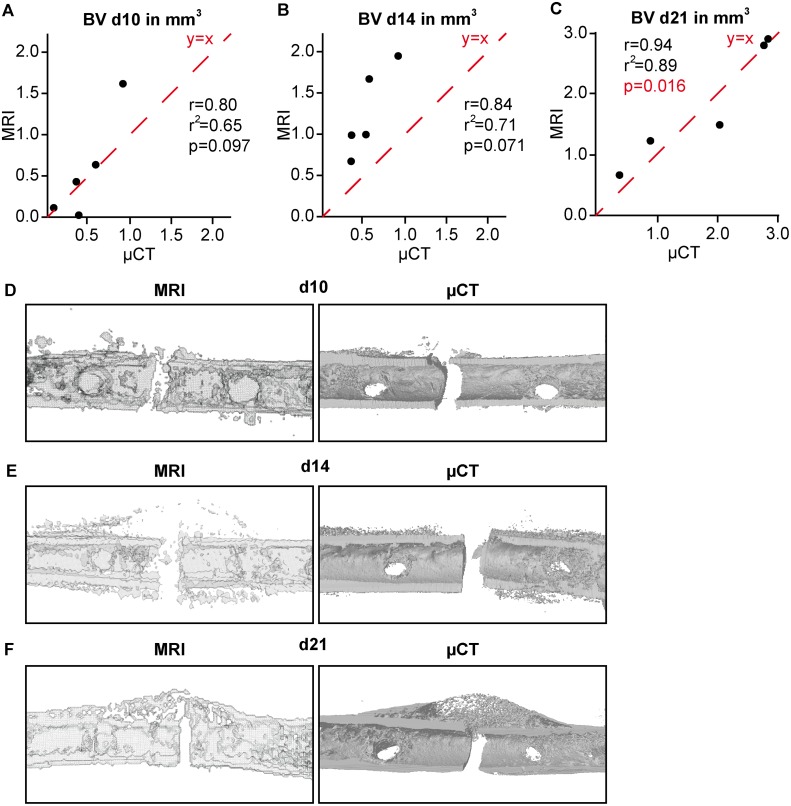
3D analysis of the bone volume (BV) in the fracture callus as determined by MRI and μCT. A) On day 10, B) on day 14 and C) on day 21. The red line y = x represents the ideal correlation between the values. D-F) 3D MRI and μCT reconstructions of the bony tissue of a fractured femur 10, 14 and 21 days after surgery. Because the MRI scan was performed *in vivo*, the fixator pins are visible. μCT scan was performed *ex vivo* and the two inner fixator pins were removed prior to the scan, therefore, holes are visible in the reconstruction.

Direct comparison of μCT and MRI data (Fig 3) did not reveal any significant differences in the bone volume measured on days 10 and 21 post-fracture, whereas MRI-derived bone volumes were significantly overestimated in the fracture callus on day 14 (Table 1). Statistical correlation analysis of the data obtained by μCT and MRI displayed high correlation values between the two methods (d10: r = 0.80, r^2^ = 0.65; d14: r = 0.84, r^2^ = 0.71; d21: r = 0.94, r^2^ = 0.89), however, this was only statistically significant on day 21 (p = 0.016).

**Table 1:** Mean and standard deviation of the bone volume in mm^3^ determined by MRI and μCT.

	d10	d14	d21
**MRI**	0.55 ± 0.66	1.29 ± 0.56*	1.82 ± 0.98
**μCT**	0.40 ± 0.26	0.53 ± 0.26	1.78 ± 1.06

*p<0.05

### Comparison of 2D data obtained by histomorphometry and MRI

**Fig 4:**
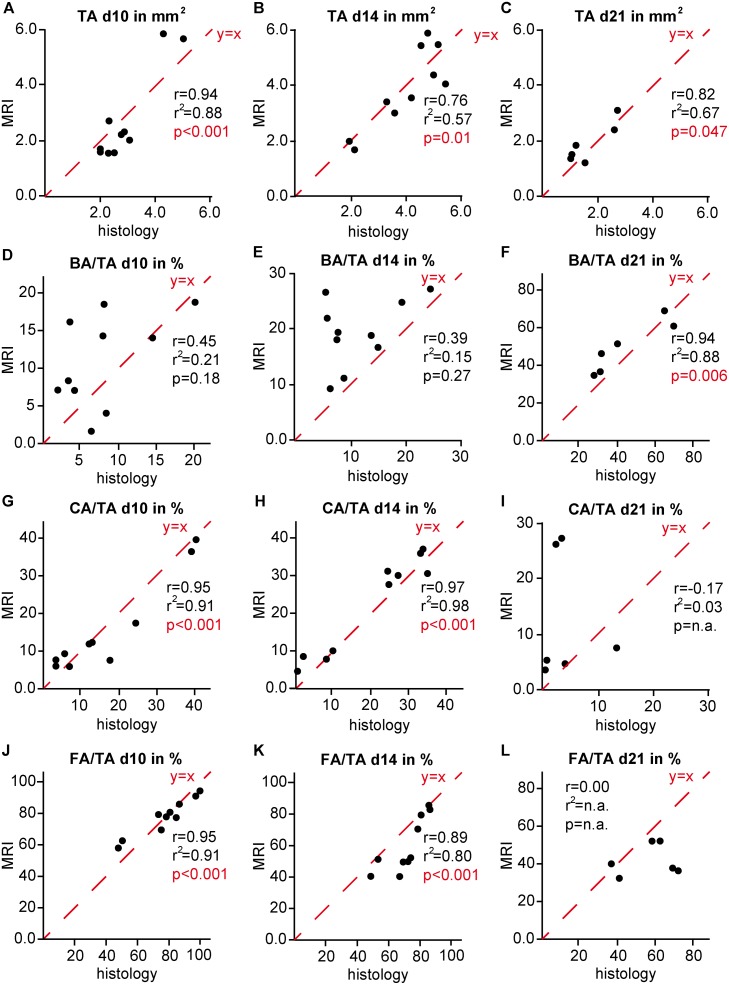
2D analysis of the total callus area (TA), relative bone area (BA/TA), relative cartilage area (CA/TA) and relative fibrous tissue area (FA/TA) in the fracture callus as determined by MRI and histomorphometry A, D, G, J) on day 10, B, E, H, K) on day 14 and C, F, I, L) on day 21. The red line y = x represents the ideal correlation between the values.

**Fig 5:**
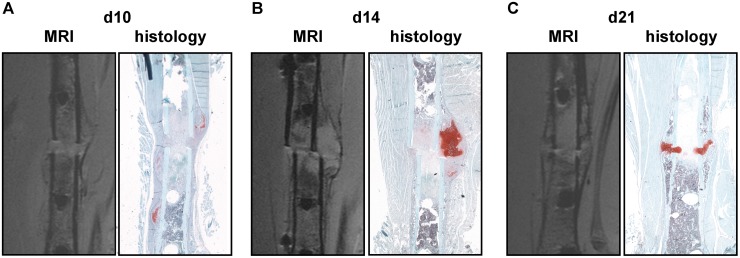
Corresponding 2D MRI and μCT slices of a fractured femur. A) On day 10, B) on day 14 and C) on day 21 after surgery. Histological sections were stained using Safranin O.

Direct comparison of histomorphometry and MRI data (Figs 4 and 5) did not reveal any significant differences in the total callus area determined during the entire fracture-healing period (Table 2). Statistical correlation analysis showed significant correlation values between the two methods at all time points (d10: r = 0.94, r^2^ = 0.88, p<0.001; d14: r = 0.76, r^2^ = 0.57, p = 0.01; d21: r = 0.82, r^2^ = 0.67, p = 0.047). Additionally, there were no significant differences in the bone fraction values determined on days 10 and 21, whereas MRI measurement significantly overrated the bone fraction on day 14 (Table 2). Bone fraction values were weakly comparable between histomorphometry and MRI on days 10 and 14 after fracture (d10: r = 0.45, r^2^ = 0.21; d14: r = 0.39, r^2^ = 0.15), but correlated significantly on day 21 (r = 0.94, r^2^ = 0.88, p = 0.006). Cartilage fraction values did not differ significantly between both methods at all time points (Table 2). In contrast to the bone fraction, cartilage fraction values were weakly comparable between histomorphometry and MRI on day 21 (r = -0.17, r^2^ = 0.03), but correlated significantly on days 10 and 14 (d10: r = 0.95, r^2^ = 0.91, p<0.001; d14: r = 0.97, r^2^ = 0.98, p<0.001). There were no significant differences in the fibrous tissue fraction values determined by both methods during the entire fracture-healing period (Table 2). Fibrous tissue fraction values displayed significant correlation on days 10 and 14, but were weakly comparable at day 21 (d10: r = 0.95, r^2^ = 0.91, p<0.001; d14: r = 0.89, r^2^ = 0.80, p<0.001; d21: r = 0.00, r^2^ = n.a.).

**Table 2:** Mean and standard deviation of the total callus area (TA) in mm^2^ and the relative bone (BA/TA), cartilage (CA/TA) and fibrous tissue areas (FA/TA) in % determined by MRI and histomorphometry.

	d10	d14	d21
*TA*			
**MRI**	2.51 ± 1.54	3.70 ± 1.31	1.83 ± 0.57
**histology**	2.69 ± 0.95	3.84 ± 1.05	1.70 ± 0.55
*BA/TA*			
**MRI**	10.84 ± 5.66	19.04 ± 5.76*	47.84 ± 10.01
**histology**	7.79 ± 5.43	11.28 ± 5.66	43.10 ± 13.55
*CA/TA*			
**MRI**	15.20 ± 11.49	20.86 ± 11.55	12.61 ± 10.64
**histology**	16.66 ± 12.75	18.27 ± 11.51	3.38 ± 4.47
*FA/TA*			
**MRI**	73.96 ± 9.20	60.10 ± 16.39	39.55 ± 6.24
**histology**	75.55 ± 13.79	70.45 ± 12.10	53.52 ± 13.53

*p<0.05

### Impact of repeated MRI on fracture healing

**Fig 6:**
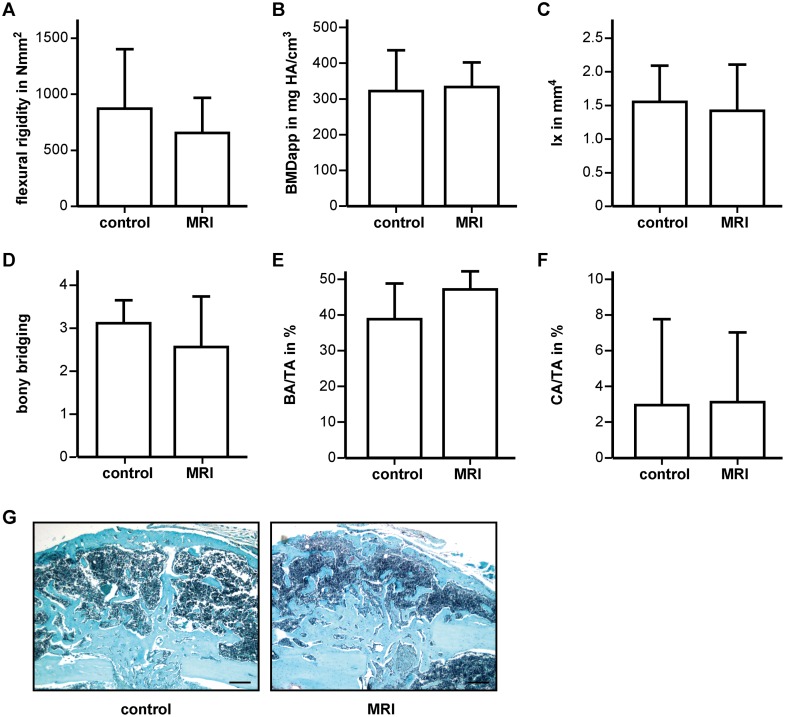
Repeated MRI scans did not disturb fracture healing. Biomechanical, μCT and histomorphometric analysis on day 28 revealed no significant differences between the groups regarding A) flexural rigidity of the fractured femur, B) apparent bone mineral density, C) moment of inertia and D) bony bridging of the fracture callus as well as in E) relative bony and F) cartilage tissue content. G) Representative histological sections from the fractured femurs stained with Safranin O. Scale bar: 200 μm.

Biomechanical analysis demonstrated that the flexural rigidity of the fracture femurs on day 28 was similar for control animals and repeatedly scanned mice (Fig 6A). μCT analysis revealed no significant differences between the groups regarding apparent bone mineral density, moment of inertia or bony bridging of the fracture callus (Fig 6B–6E). Confirming this, histomorphometric analysis did not show altered bone or cartilage tissue areas in scanned mice compared to control mice (Fig 6E–6G).

## Discussion

The aim of this study was to evaluate high-resolution *in vivo* MRI for the longitudinal assessment of the different tissues in the fracture callus during bone healing in mice. We established algorithms for tissue characterization based on the intensity of the MRI signal and correlated the values with those obtained by the two *ex vivo* gold standard methods μCT and histomorphometry. We observed that the accuracy of the MRI measurements was highly dependent on the size of the measured tissue. Higher relative tissue area values resulted in a better correlation between MRI, μCT and histomorphometry. Importantly, we also demonstrated that repeated anesthesia, handling and MRI scans did not disturb bone healing. Therefore, this study demonstrated the feasibility of high-resolution *in vivo* MRI for longitudinal assessment of soft callus formation during the early and intermediate phases of fracture healing in mice.

To date, only two studies reported applying MRI during bone regeneration in mice. Zachos et al. [9] compared high-detail radiography, helical CT and high-field MRI to histology during articular fracture healing in mice. The study demonstrated that only histology was a reliable tool to determine the articular fracture-healing progress in the mouse model because of the low resolution of the other tested methods. However, the limitations of that study are firstly, that the mice were sacrificed before the MRI examination and therefore the tissue contrast may have differed from the *in vivo* situation, and secondly, the limited spatial resolution possible with the 4.7-Tesla system used in this study. Taha et al. [10] investigated different MR imaging techniques at 9.4-T field strength and showed that a proton density weighted TSE sequence performed best for the evaluation of bone healing. This method was able to discriminate between bone and soft tissue, including the medullary space and granulation tissue at the injury site at a higher contrast than μCT imaging. However, a drill-hole bone-defect model in the mouse tibia was used in this study, which does not result in endochondral bone healing with the formation of a cartilaginous callus. None of the studies evaluated the impact of repeated MRI scans on the bone-healing process.

Based on the longitudinal scans during the fracture-healing period, we established algorithms for tissue characterization based on the intensity of the MRI signal. For evaluation of the accuracy of the measured values, we directly compared the 3D bone volumes between the 3D gold standard μCT and MRI as well as 2D tissue fractions in the fracture callus between the 2D gold standard histomorphometry and MRI. We demonstrated that MRI data were weakly comparable to μCT and histomorphometry for small tissue values. This might be due to the limited spatial resolution of MRI. μCT scanning was performed using an isotrophic resolution of 8 μm, whereas MRI voxel size was 52×52×350 μm. Therefore, the spatial resolution of MRI is 6.5-times less than the resolution of μCT in x and y direction and even 43.75-times less in z direction. This lower spatial resolution is a strong limitation of the MRI method when compared to competing techniques, including μCT. Importantly, however, at the early and intermediate fracture-healing phases, when the cartilaginous callus formation peaked, fibrous tissue and cartilage values were highly accurate. Because the amount of cartilage tissue in the early fracture callus provides important information on fracture-healing progress [4], using the *in vivo* MRI technique for longitudinal analysis of endochondral fracture healing might improve the monitoring of future murine fracture-healing studies and may improve our knowledge of soft callus development.

Additionally, our results demonstrated that repeated scans did not impact the fracture-healing process, thus proving a negligible impact of the likely decrease of the level of inflammatory cytokines due to the halothanes used for anesthesia [15] and the increased stress to the animal during scanning. Even though physiological changes, including increased permeability of the blood-brain barrier [16], decreased red blood cell membrane permeability [17], a possible magnetic field-induced heating of tissues [18] and a transient increase in DNA damage after exposing lymphocytes to magnetic fields *in vitro* [19], have been reported, no impact on fracture healing was observed in this study, thus providing the basis for the further use of this *in vivo* technique.

In conclusion, this study demonstrated the feasibility and accuracy of high-resolution *in vivo* MRI for longitudinal analysis of soft callus formation during the early and intermediate phases of fracture healing in mice. Future perspectives for the use of MRI during murine bone-healing studies are a possible combination of MRI scans with the use of contrast agents, magnetic-labeled cells or a PET system. These applications would allow the measurement of the blood flow and oxygen consumption in the fractured leg as well as cell trafficking through fluorescence or SPIO (superparamagnetic particles of iron oxide) labeling as it was shown for other disease models [20–23].
